# Pseudo-spontaneous nystagmus in patients with geotropic direction-changing positional nystagmus

**DOI:** 10.1371/journal.pone.0196019

**Published:** 2018-04-23

**Authors:** Seok Min Hong, Sung Kyun Kim, Il-seok Park, Min Gyeong Shim

**Affiliations:** Department of Otorhinolaryngology-Head and Neck Surgery, Dongtan Sacred Heart Hospital, Hallym University College of Medicine, Hwaseong, Korea; Yokohama Brain and Spine Center, JAPAN

## Abstract

**Background:**

Pseudo-spontaneous nystagmus has been reported in patients with direction-changing positional nystagmus (DCPN). Recently, the concept of a “light cupula” has been introduced as a pathophysiology that can exhibit persistent geotropic DCPN. Patients with persistent DCPN could have different characteristics of nystagmus. Therefore, we investigated the pseudo-spontaneous nystagmus in patients with transient (canalolithiasis) and persistent (belong to light cupula theory) geotropic DCPN.

**Methods:**

In this study, prospectively, 49 patients with persistent geotropic DCPN and 67 patients with transient geotropic DCPN were enrolled. We compared the incidence of pseudo-spontaneous nystagmus between persistent and transient DCPN patients and characteristics of pseudo-spontaneous nystagmus and positional nystagmus by the head roll test in these patients. A prospective study was conducted at a dizziness clinic.

**Results:**

Patients with persistent geotropic DCPN exhibited significantly higher incidence of pseudo-spontaneous nystagmus than patients with transient geotropic DCPN. Patients with transient DCPN showed a significantly higher mean SPV value during the head roll test than patients with persistent DCPN. All patients exhibiting pseudo-spontaneous nystagmus in patients with persistent DCPN had a null plane, and all patients had nystagmus beats to the opposite side of the null plane or the lesion side.

**Conclusion:**

Our results support the possibility that the mechanism between persistent and transient geotropic DCPN may be different. However, more studies are needed on the pathogenesis and mechanism of the two diseases, including the occurrence of pseudo-spontaneous nystagmus in the disease entity.

## Introduction

Benign paroxysmal positional vertigo (BPPV) is characterized by short-lasting vertigo that develops when the dependent position of the head changes [1]. BPPV is explained by the theory of free-floating otolithic debris in the endolymph of the semicircular canal (canalolithiasis) or debris near or attached to the cupula (cupulolithiasis) [2].

Direction-changing positional nystagmus (DCPN) is typically observed in patients with lateral semicircular canal BPPV when the head is turned to either side in the supine position. In canalolithiasis-type lateral semicircular canal-BPPV, the DCPN beats towards the lowermost ear [3].

Several recent studies have reported the occurrence of spontaneous nystagmus in the sitting position in patients with lateral semicircular canal-BPPV [4–6]. Because the spontaneous nystagmus is observed without imbalance in bilateral vestibular tone that usually develops in acute unilateral vestibulopathy, it has been named ‘pseudo-spontaneous nystagmus’, which was possibly associated with BPPV and not provoked by positioning but not yet sufficiently understood, formulated by the Committee for Classification of Vestibular Disorders of the Bárány Society [7].

Also, recently, the concept of a “light cupula” in the lateral semicircular canal, revealing a relatively long-duration geotropic (persistent) DCPN that was different from typical transient geotropic DCPN (canalolithiasis), has been introduced as a variant of lateral semicircular canal-BPPV [3,8–10]. We thought that the pathogenesis of persistent DCPN may not originate from free-floating debris but from deflection of the cupula [10], and patients with persistent geotropic DCPN could have different characteristics of nystagmus compared to patients with transient geotropic DCPN.

In the present study, we investigated the spontaneous nystagmus in the patients with persistent and transient geotropic DCPN, and we examined the difference in spontaneous nystagmus incidence and compared the characteristics of nystagmus between two groups.

## Materials and methods

The study was approved by the Institutional Review Board of Hallym Dongtan Sacred Heart Hospital(2015-518-I). Forty-nine patients with persistent geotropic DCPN and Sixty-seven with transient geotropic DCPN seen between December 2015 and September 2017 were investigated. All patients underwent detailed medical history-taking and audiological and vestibular evaluations, including auditory evoked potential, video head impulse test, and caloric test. We tried to exclude all possible diseases that could cause DCPN. Patients with other labyrinthine diseases, recent head trauma, sudden hearing loss, recent surgery, or concomitant otologic pathology were excluded.

Neurological examination revealed no abnormality in any of the patients. Eye movement was examined in various head positions and recorded using goggles fitted to an infrared camera (Micromedical, Chatham, IL; SLMED, Seoul, Korea). Nystagmus was documented using a video-based system (Micromedical Technologies Inc., Chantham) in some patients in order to calculate the slow-phase velocity (SPV) of the horizontal nystagmus.

All patients were assessed for the presence of spontaneous nystagmus in the sitting position, and underwent in the order of head roll test and Dix-Hallpike test after observing spontaneous nystagmus. Spontaneous horizontal nystagmus was observed for at least 30 seconds without any head movement and mean SPV values during 10 second were measured for right and left head turns in the head-roll test.

The diagnostic criteria for persistent DCPN were the presence of geotropic DCPN for over 2 min after a supine head-roll test and the presence of a null plane [3]. Patients with transient DCPN had typical BPPV with geotropic DCPN for less than 1 min.

Lateralization of the patients with transient DCPN was based on the stronger side of positional nystagmus during the head roll test, and lateralization of patients with persistent DCPN was based on identification of a null plane and the head roll test.

We compared the incidence rate of spontaneous nystagmus (pseudo-spontaneous nystagmus) between patients with transient and persistent DCPN, and examined the clinical characteristics of patients with pseudo-spontaneous nystagmus. We also compared the mean SPV during the head roll test between the two groups.

### Statistical analysis

Clinical parameters, pseudo-spontaneous nystagmus incidence rate, and mean SPV in the two groups were compared using an independent *t*-test or the chi-squared test. All statistical analyses were conducted using SPSS version 17.0 (SPSS/PC, Chicago IL), with statistical significance set at *p <* 0.05.

## Results

The demographic and clinical data of groups with transient and persistent geotropic DCPN are summarized in Table 1. The two groups were well matched in terms of age, sex ratio, the affected side, and duration of symptom, and the parameters did not differ significantly between the two groups.

**Table 1:** Demographic findings between patients with transient and persistent geotropic DCPN(direction changing positional nystagmus).

	Transient DCPN	Persistent DCPN
Age,y, mean age SD(range)	48.9±16.4(27–93)	49.9±13.1(26–76)
Sex ratio:men/women(n)	17:50	17:32
Affected side: right/left(n)	32:35	29:20
Duration of symtom, days	3.1±4.4	3.9±3.9

**Fig 1:**
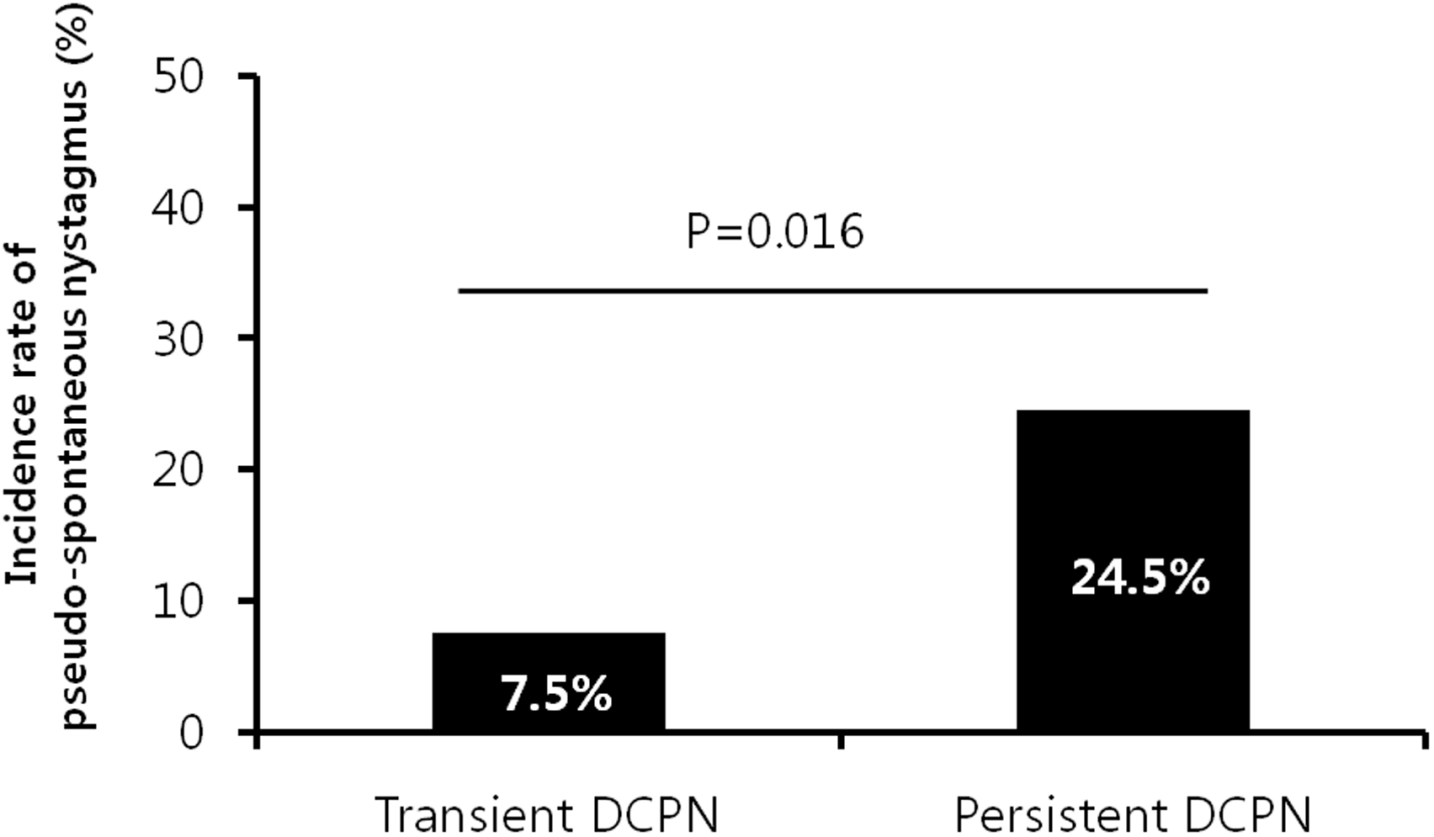
Comparison of pseudo-spontaneous nystagmus incidence between transient and persistent geotropic DCPN(direction-changing positional nystagmus) patients.

Pseudo-spontaneous nystagmus in the sitting position was observed in 5 (7.5%) of the 67 patients with transient DCPN and in 12 (24.5%) of the 49 patients with persistent DCPN, and patients with persistent DCPN showed significantly higher incidence of pseudo-spontaneous nystagmus (p = 0.016; Fig 1).

Of the five patients with pseudo-spontaneous nystagmus in the transient DCPN group, four patients had spontaneous nystagmus in the opposite direction as the lesion, on patient had spontaneous nystagmus in the same direction as the lesion. They did not exhibit the null plane. All 12 patients exhibiting pseudo-spontaneous nystagmus in the persistent DCPN group had a null plane by definition, and the SPV of pseudo-spontaneous nystagmus ranged from 1 to 8 deg/sec and all patients had nystagmus beats to the opposite side of the null plane or the lesion side (Table 2).

**Table 2:** Clinical characteristics of the patients with pseudo-spontaneous nystagmus(PSN) in patients with transient and persistent DCPN(direction-changing positional nystagmus).

Group	Age	Sex	Lesion side	PSN SPV/direction	Head Roll SPV/direction	Null plane
Canalolithiasis	28	F	L	4/RB	82/LB	none
	43	M	L	3/LB	75/LB	none
	77	F	R	5/LB	89/RB	none
	93	F	L	5RB	56/LB	none
	70	M	R	4LB	36/RB	none
Light cupula	52	F	R	1/LB	19/RB	R
	34	M	R	1/LB	27/RB	R
	44	M	R	8/LB	30/RB	R
	45	M	L	2/RB	11/LB	L
	76	M	L	2/RB	34/LB	L
	48	F	R	3/LB	14/RB	R
	41	F	R	3/LB	25/RB	R
	26	F	L	3/RB	22/LB	L
	63	M	R	3/LB	18/RB	R
	57	F	R	2/LB	38/RB	R
	57	F	R	3/LB	28/RB	R
	39	F	R	3/LB	11/RB	R

PSN: Pseudo-Spontaneous Nystagmus, SPV: Slow-Phase Velocity, M: male, F: female, R: right, L: Left, Head roll SPV/direction: Slow-Phase Velocity and direction of nystagmus during head roll test toward lesion side.

**Fig 2:**
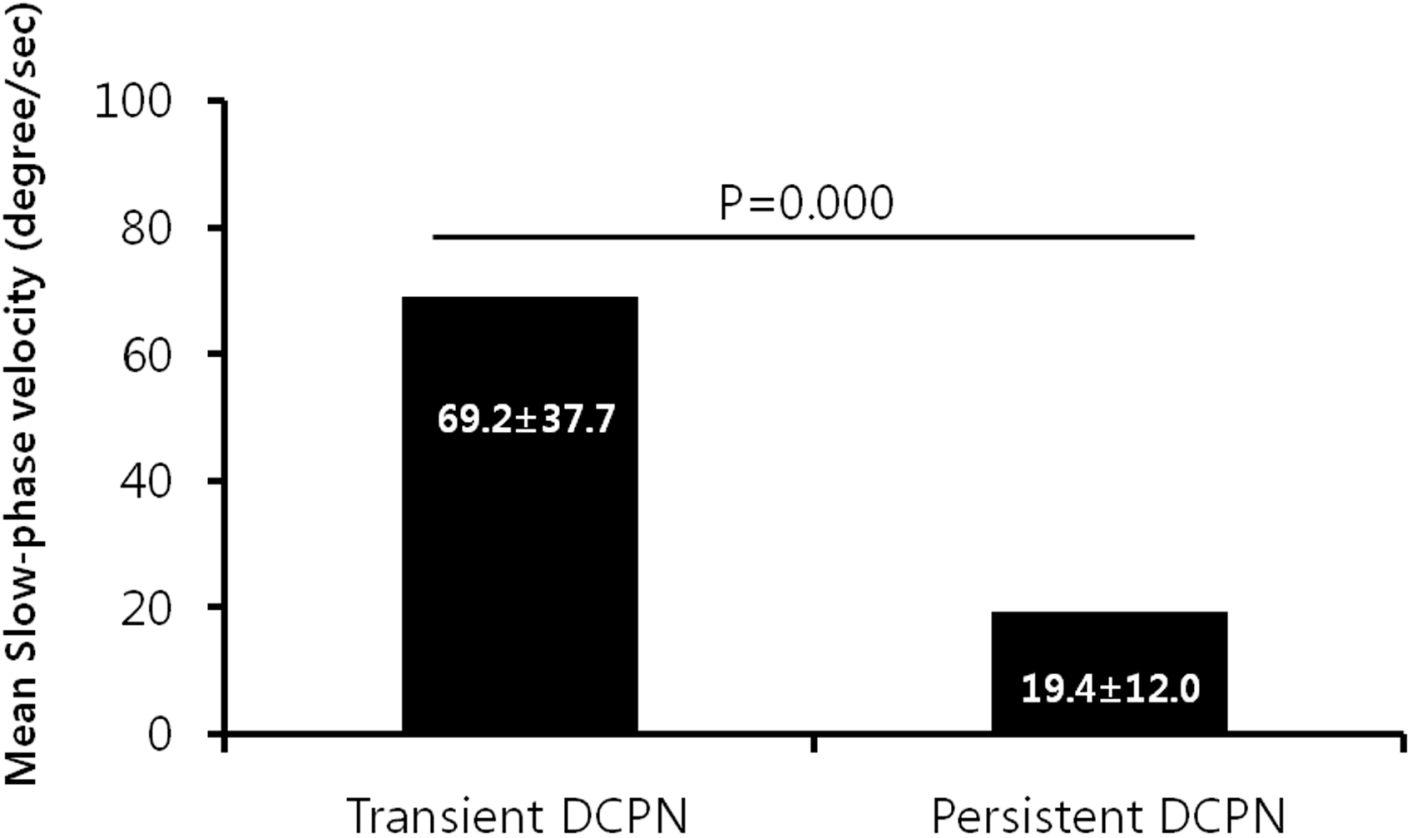
Comparison of mean slow-phase velocity in head roll test between transient and persistent geotropic DCPN(direction-changing positional nystagmus) patients.

Mean SPV values in the lesion side of patients with transient and persistent DCPN during the head roll test were 69.2±37.7 deg/sec and 19.4±12.0 deg/sec, respectively, and this difference was statistically significant (p = 0.000, Fig 2).

## Discussion

The occurrence of spontaneous nystagmus in DCPN has been explained by two pathophysiologic mechanisms. One is ‘otolithic jam theory’ that induces plugging of the narrow semicircular canals, but it is very rare [11]. The other is pseudo-spontaneous nystagmus whose reported incidence is widely variable (14.1% -68.5%) [5,6,12]. This incidence can vary according to the examination time, head position of patients [6] and the standards of spontaneous nystagmus such as intensity and duration.

In this study, we have focused on pseudo-spontaneous nystagmus in geotropic DCPN, excluding spontaneous nystagmus with a otolithic jam and apogeotropic DCPN. Geotropic DCPN that is persistent and without latency or fatigability has been explained as a condition of light cupula [2,8,10], which occurs when the specific gravity of the cupula is lower than the specific gravity of the surrounding endolymph.

The lateral semicircular canal has a 30-degree anterior inclination from the horizontal plane when the patients are placed in a sitting position. Until now, the cause of pseudo-spontaneous nystagmus in patients with geotropic DCPN has been explained by the slow movement of otoliths across the inclined lateral semicircular canal [4]. The occurrence and direction of pseudo-spontaneous nystagmus in geotropic DCPN would depend on the position of the otolithic debris in the lateral semicircular canal before assuming the head upright position and the direction of pseudo-spontaneous nystagmus could be either ipsilesional or contralesional [5].

**Fig 3:**
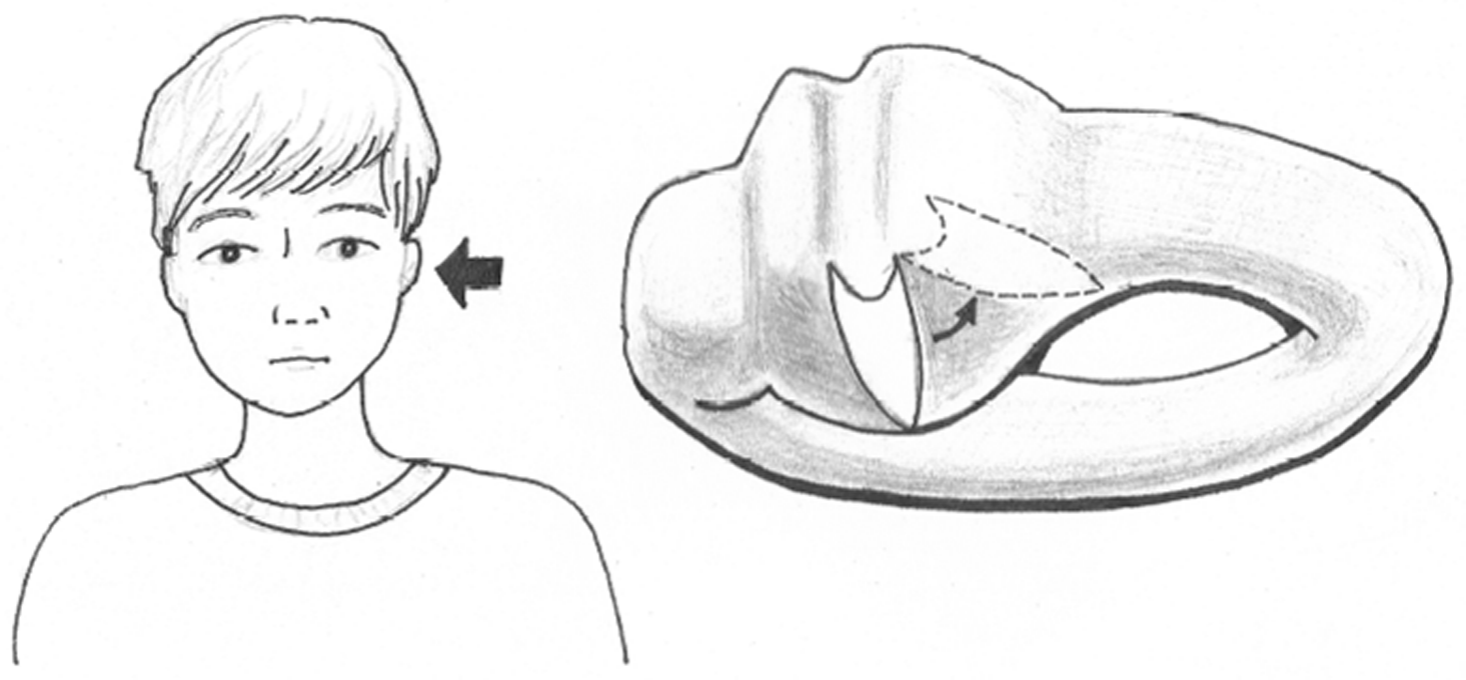
Theoretical findings of spontaneous nystagmus in representative cases of persistent geotropic DCPN(direction-changing positional nystagmus) in the left lateral semicircular canal(the cupula is ampulopetally deviated in sitting position).

We detected the occurrence of pseudo-spontaneous nystagmus in patients with persistent geotropic DCPN. The pathogenesis of persistent geotropic DCPN might originate from deflection of the cupula [10]. Therefore, the mechanism of pseudo-spontaneous nystagmus could be explained by the upward deflection (toward the ampullofugal direction) of the cupula that is tilted in the sagittal plane with its base pointing superiorly and medially in the sitting position (Fig 3) [10]. In this study, patients with persistent DCPN exhibited a 24.5% pseudo-spontaneous nystagmus incidence rate in contrast to patients with transient DCPN who exhibited a pseudo-spontaneous nystagmus incidence rate of only 7.5%. In patients with transient DCPN, the position of otoliths near either ends of the semicircular canals for endolymphatic flow is necessary for the occurrence of pseudo-spontaneous nystagmus, but in patients with persistent geotropic DCPN, slight head movement would be enough to induce pseudo-spontaneous nystagmus if only the density of the cupula is lower than that of the endolymph.

The direction of pseudo-spontaneous nystagmus was the same or to the opposite side of the lesion side in patients with transient DCPN, but, the direction of pseudo-spontaneous nystagmus was to the opposite side of the lesion or the null plane in all patients with persistent DCPN. As we have mentioned above, in the sitting position, because the cupula is deflected to the canal side and it induces ampullofugal endolymphatic flow, pseudo-spontaneous nystagmus may occur in the opposite direction of the lesion side. These findings were thought to support the pathophysiology of deflection of the cupula.

In this study, we found that that there was a difference in mean SPV during the head roll test between patients with transient and persistent DCPN. The SPVs recorded in patients with transient DCPN probably represent many factors influencing nystagmus velocity, including the volume of otoconia, the endolymph viscosity and the order of testing [13]. Considering that the mechanism of persistent geotropic DCPN as deflection of the cupula, SPVs in patients with persistent DCPN would reflect the relative density of the cupula to the endolymph viscosity, but the variation of the relative density in these patients would be less than the variation in otoconia volume in patients with transient DCPN, which originate from free-floating otolith. This means that there would be a limit to the increase beyond a certain SPV value in patients with persistent DCPN.

## Conclusion

Pseudo-spontaneous nystagmus was observed more frequently in patients with persistent DCPN than in patients with transient DCPN. There was also a difference in the mean SPV during head roll test between the two groups. Our results support the possibility that the mechanism between persistent and transient geotropic DCPN may be different. However, more studies are needed on the pathogenesis and mechanism of the two diseases, including the occurrence of pseudo-spontaneous nystagmus in the disease entity.

## Supporting information

S1 File Dataset of patients included in this study. (XLSX)
